# Superordinate Level Processing Has Priority Over Basic-Level Processing in Scene Gist Recognition

**DOI:** 10.1177/2041669516681307

**Published:** 2016-12-06

**Authors:** Qi Sun, Yanju Ren, Yang Zheng, Mingxia Sun, Yuanjie Zheng

**Affiliations:** School of Psychology, Shandong Normal University, Jinan, P. R. China; Department of Psychology, Zhejiang Normal University, Jinhua, P. R. China; School of Psychology, Shandong Normal University, Jinan, P. R. China; Department of Psychology, Zhejiang Normal University, Jinhua, P. R. China; School of Public Administration, Shandong Normal University, Jinan, P. R. China; School of Information Science and Engineering, Shandong Normal University, Jinan, P. R. China; Perelman School of Medicine, University of Pennsylvania, PA, USA; Institute of Life Sciences, Shandong Normal University, Jinan, P. R. China; Key Laboratory of Intelligent Information Processing, Shandong Normal University, Jinan, P. R. China

**Keywords:** visuospatial working memory load, scene gist recognition, superordinate level, basic level, hierarchical processing

## Abstract

By combining a perceptual discrimination task and a visuospatial working memory task, the present study examined the effects of visuospatial working memory load on the hierarchical processing of scene gist. In the perceptual discrimination task, two scene images from the same (manmade–manmade pairing or natural–natural pairing) or different superordinate level categories (manmade–natural pairing) were presented simultaneously, and participants were asked to judge whether these two images belonged to the same basic-level category (e.g., street–street pairing) or not (e.g., street–highway pairing). In the concurrent working memory task, spatial load (position-based load in Experiment 1) and object load (figure-based load in Experiment 2) were manipulated. The results were as follows: (a) spatial load and object load have stronger effects on discrimination of same basic-level scene pairing than same superordinate level scene pairing; (b) spatial load has a larger impact on the discrimination of scene pairings at early stages than at later stages; on the contrary, object information has a larger influence on at later stages than at early stages. It followed that superordinate level processing has priority over basic-level processing in scene gist recognition and spatial information contributes to the earlier and object information to the later stages in scene gist recognition.

When watching television, we can grasp some important information (e.g., an actor's emotion and dress, or the type of program: cartoon vs. epic) with just a glance, regardless of how quickly the channels flip. These kinds of information were described as scene gist in the domain of scene perception. Scene gist recognition was operationally defined in numerous ways, but usually in terms of the ability to classify a briefly flashed scene image at some level of abstraction (Larson, Freeman, Ringer, & Loschky, 2014). Potter (1976) found that the accuracy of scene gist recognition was above 70% with just 113 ms presentation in a rapid serial visual presentation sequence, indicating scene gist recognition was a kind of very efficient cognitive ability. Many investigators have examined scene gist recognition from different perspectives, such as human behavior experiments (Banno & Saiki, 2015; Delorme, Richard, & Fabre-Thorpe, 2010; Greene & Fei-Fei, 2014; Kadar & Ben-Shahar, 2012; Larson et al., 2014), animal studies (Kirkpatrick, Bilton, Hansen, & Loschky, 2014), computational studies (Fei-Fei & Perona, 2005; Oliva & Torralba, 2001; Xiao, Ehinger, Hays, Torralba, & Oliva, 2014), eye movements recordings (Malcolm, Nuthmann, & Schyns, 2014; Wu, Crouzet, Thorpe, & Fabre-Thorpe, 2014), event-related potentials recording (Bacon-Macé, Macé, Fabre-Thorpe, & Thorpe, 2005; Codispoti, Ferrari, Junghöfer, & Schupp, 2006; Groen, Ghebreab, Prins, Lamme, & Scholte, 2013), functional magnetic resonance imaging (fMRI) studies (Peelen, Fei-Fei, & Kastner, 2009; Ramkumar et al., 2014; Walther, Chai, Caddigan, Beck, & Fei-Fei, 2011), and gene extraction techniques (Kikuno, Matsunaga, & Saiki, 2013).

Although many efforts have been made on the mechanism of scene gist recognition, a great number of questions about visual and cognitive processes in scene gist recognition remain unclear. Previous studies have pointed out that scene gist includes three different levels of categories: superordinate (e.g., manmade vs. natural), basic (e.g., bedroom vs. city center), and subordinate (e.g., college classroom vs. elementary classroom) levels, and some studies have demonstrated that processing of scene gist was hierarchical (Fei-Fei, Iyer, Koch, & Perona, 2007; Kadar & Ben-Shahar, 2012; Tversky & Hemenway, 1983). One important question is the order of hierarchical processing in scene gist recognition, that is, it is unclear whether the superordinate level is prior to the basic level or not.

For the question mentioned above, basic-level superiority and superordinate level superiority were proposed. Tversky and Hemenway (1983) provided three groups of participants with scene names from different categories and asked participants to list their attributes. The results showed that the number of attributes listed by the basic-level group was larger than in the superordinate level group. Hence, they concluded that the processing of basic level was prior to that of superordinate level.

However, recent findings have challenged this view and thought that the processing of superordinate level was prior to that of basic level. Schyns and Oliva (1994) first used Fourier filter to extract the low frequency (holding global information of scene images, such as, general orientations and proportions) and high frequency (representing abrupt spatial changes in images, and generally corresponding to configural information and fine details) components of two different images, then combined these two components to make a hybrid image. In each trial, a hybrid image was presented for a short (30 ms) or long (150 ms) duration; after the presentation, participants were required to make a judgment as to whether the hybrid image matched the pre-specified target or not. The results revealed that when the hybrid images were presented for 30 ms, participants made decisions based on low-frequency components; however, when the presentation time was 150 ms, participants made judgments according to high-frequency components. Based on these results, Schyns and Oliva proposed that the processing of scene gist was from coarse to fine. Kadar and Ben-Shahar (2012) adopted category discrimination task to explore the hierarchical processing of scene gist. In each trial, two scene images were presented simultaneously and participants were asked to make a judgement whether they belonged to the same basic-level category or not. The results of both multidimensional scaling (MDS) analysis and temporal dynamics revealed that processing of superordinate level was prior to that of basic level. This supported the priority of superordinate level over basic level directly. However, recent investigators found that the order of hierarchical processing in scene gist recognition was unstable, depending on different categories and category structures (Banno & Saiki, 2015).

Relevant to the order of hierarchical processing in scene gist recognition, the roles of spatial and object information processing in scene gist recognition also have become an important focus. Specifically speaking, whether both of them are equally important in scene gist recognition and whether spatial information takes priority over object information or not.

Oliva and Torralba (2001) proposed a spatial envelop model, in which observers recognized scene gist according to five spatial envelop properties: naturalness, openness, roughness, expansion and ruggedness, and did not need to recognize objects in scene images. First, participants classified scene images into a natural or manmade category according to naturalness, and then made a finer classification (e.g., kitchen, city). This study revealed that the holistic information (especially spatial information), rather than local information (mainly object information), had an early influence on scene gist recognition and this result was also replicated in other studies (e.g., Fei-Fei et al., 2007; Kadar & Ben-Shahar, 2012). However, Greene (2013) first used the LabelMe toolbox (Russell, Torralba, Murphy, & Freeman, 2008) to annotate objects in scene images, and then used the linear classifier to classify the scene images with the labeled objects. The results showed that the labeled objects had an important influence on the scene images classification. Some fMRI experiments also found that object-selective visual cortex, often referred to as the lateral occipital complex (LOC), was activated in scene recognition (Peelen et al., 2009; Walther, Caddigan, Fei-Fei, & Beck, 2009). These studies suggested that local information impacts on scene recognition.

More and more studies revealed that both local and global information had influences on scene gist recognition. For example, using fMRI technology to record the brain activity, MacEvoy and Epstein (2011) found that the parahippocampal place area and LOC were both activated when participants recognized scene gist. Meanwhile, Kravitz, Peng, and Baker (2011) further revealed that parahippocampal place area was responsible for the processing of spatial information, especially the openness and expansion, and that early visual cortex, related to the processing of deepness, was also activated. Thus, both spatial and object (nonspatial) information contribute to scene gist recognition.

Recently, the interaction between visual perception and working memory has become a key topic in cognitive psychology (e.g., Konstantinou & Lavie, 2013; Soto, Wriglesworth, Bahrami-Balani, & Humphreys, 2010). Working memory plays a great role in complex cognitive activities, like visual perception, language comprehension, learning, and reasoning. According to Baddeley's multicomponents model, working memory consists of four subsystems: visuospatial sketchpad, episodic buffer, phonological loop and central executive, and the visuospatial sketchpad could be further divided into two sub-components: spatial working memory and object working memory. The two sub-components are mainly responsible for the processing and maintenance of spatial and object information, respectively (for a review, see Baddeley, 2012). Scene gist recognition is an important activity of visual perception, and the exploration of the interaction between scene gist recognition and different components of working memory could help us understand the processing mechanisms of spatial and nonspatial information and the order of hierarchical processing in scene gist recognition.

In the present study, we designed two experiments to explore the effects of visuospatial working memory on hierarchy in scene gist processing using a dual-task paradigm in which participants performed a perceptual discrimination task while maintaining spatial or object information in working memory. In the perceptual discrimination task, two scene images from the same or different superordinate level categories were presented simultaneously and participants were asked to judge whether these two images belonged to the same basic-level category or not. In the spatial working memory task (Experiment 1), before performing the perceptual discrimination task, participants were required to remember the positions of no or four squares and after the perceptual discrimination task, participants were instructed to judge whether the position of a probe square was present or not. In the same vein, in the object working memory task (Experiment 2), participants were asked to remember shapes of geometric figures and instructed to judge whether the geometric figure was present in the probe display or not.

Based on the aforementioned findings, we hypothesize the following: (a) If superordinate level processing is prior to the basic-level processing, then in perceptual discrimination task, to discriminate whether the simultaneously presented two scene images belong to the same or different basic-level category is easier when these two images are from the different superordinate level categories (manmade–natural pairing) than when these two images are from the same superordinate level categories (natural–natural or manmade–manmade pairings). Otherwise, it will be the opposite. Furthermore, as a previous study found that artificial scenes were processed slower than natural scenes (Rousselet, Joubert, & Fabre-Thorpe, 2005), we can expect the discrimination performance was lower for manmade–manmade pairing than for natural–natural pairing. (b) If superordinate level processing takes priority over the basic-level processing, then spatial and object working memory loads have less influence on discrimination of superordinate level categories than of basic-level categories. On the contrary, if basic-level processing takes priority over the superordinate level processing, then spatial and object working memory loads have less influence on discrimination of basic-level categories than of superordinate level categories. (c) During scene gist recognition, if spatial information is processed earlier than object information, then spatial working memory load should have an earlier influence on the discrimination than object working memory load. On the contrary, if object information is processed earlier than spatial information, then object working memory load should have an earlier influence on the discrimination process than spatial working memory load. Finally, if both spatial and object information have a simultaneous influence on the discrimination process, then spatial and object working memory loads have the same influence on discrimination.

## Experiment 1: Spatial Working Memory Load

In this experiment, we combined a scene gist recognition task (Kadar & Ben-Shahar, 2012) with a spatial working memory task to examine the effect of spatial working memory load on hierarchy in scene gist processing.

### Methods

#### Participants

After giving informed consent, 16 participants (15 females, one male; average age = 20.32) were paid to participate in Experiment 1. All had normal or corrected to normal vision and were naïve about the purpose of this experiment.

#### Apparatus

Stimuli were presented on a 17-in. LCD color monitor with a resolution of 1600 × 900 pixels and a refresh rate of 75 Hz. Each participant's head position was fixed by a chinrest. The experiment was developed in E-Prime 2.0 (Psychology Software Tools, Pittsburgh, PA, USA).

#### Stimuli

In this experiment, the participants sitting in a distance of 60 cm from the screen performed three tasks: articulatory suppression task, working memory task, and scene gist discrimination task.

##### Articulatory suppression task

In Experiment 1, the stimuli in this task consisted of four capital English letters. In each trial, four capital English letters (e.g., BTUG) randomly chosen from 26 English letters were presented for 1,000 ms. After a few seconds, one capital letter was shown and the participants were required to make a judgment as to whether the letter was one of the previously presented four letters or not and pressed the corresponding keys. During this task, participants were asked to repeat the sequence of four letters aloud throughout each trial to suppress verbal coding. The font of letters was bold and the color was black. Each letter subtended about 1.12° × 1.12°.

##### Working memory task

In Experiment 1, four black squares were presented on a white background. The positions of the four squares, each subtending 1.12° × 1.12°, were randomly chosen from eight assigned positions (see Table 1), which were on an imaginary circle with a 2.26° radius around the central black fixation cross subtending 0.6° × 0.6°. In each trial, four squares were presented for 500 ms. After a few seconds, another black square was shown and participants were instructed to judge whether the position of the black square was presented before or not by pressing the corresponding keys.

**Table 1.** Possible Eight Positions of Squares in Working Memory Task in Experiment 1.

	1	2	3	4	5	6	7	8
x-coordinate (°)	17.82	17.82	15.07	15.07	15.89	19.75	15.89	19.75
y-coordinate (°)	7.29	12.86	10.08	10.08	8.12	8.12	12.04	12.04

##### Scene gist recognition task

**Figure 1.**
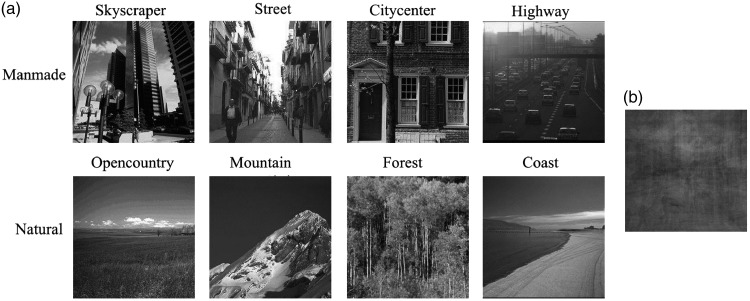
Representative example images from eight scene categories (a) and mask (b).

In each trial, two scene images were presented simultaneously for 27 ms or 507 ms (all durations are multiplies of a 75 Hz refresh cycle of the computer monitor, Fei-Fei et al., 2007; Kadar & Ben-Shahar, 2012). The gap between two images was 1.12°. After presentation, a pair of masks masked the two images for 1,000 ms. The two masks were created by averaging all scene images (Figure 1(b), subtending 5.75° × 5.75°). Then a response cue was shown on the screen that required participants to discriminate whether the paired scene images belonged to the same basic-level category or not by pressing the corresponding keys. The underlying pool of scenes used for the experiments consisted of 1,432 images from eight categories chosen from two published datasets (Fei-Fei & Perona, 2005; Oliva & Torralba, 2001): *Skyscraper, Street, Inside city, Highway, Open country, Mountain, Forest*, and *Coast* (Figure 1(a)). The first four categories were manmade, and the remaining four categories were natural. Each category contained 179 images. All images were reduced to be monochrome and resized into 5.75° × 5.75°.

### Design and Procedure

Experiment 1 lasted approximately 120 min and included three phases: Learning phase, exercise phase, and experiment phase. At the beginning of each phase, participants received instructions about which task they were to perform. In each trial of learning phase, a fixation cross lasting 500 ms was followed by a category name (e.g., mountain) for 500 ms; after the name, a scene image was shown for 1,000 ms. In this phase, the participants were required to familiarize themselves with the eight scene categories.

**Figure 2.**
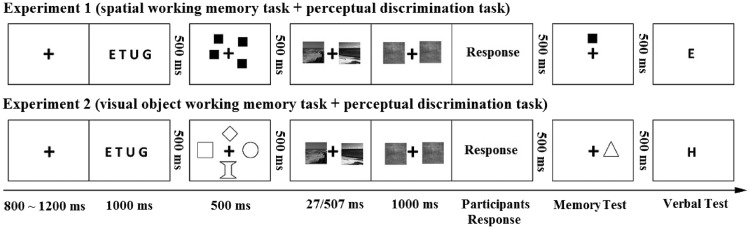
One representative trial for each dual task in Experiments 1 and 2.

The procedure of the exercise phase was the same as that of experiment phase. Figure 2 demonstrates the sequence of events of dual tasks in each trial which began with a fixation cross for 500 to 1,000 ms, followed by four capital letters (e.g., E T U G) lasting 500 ms. After 500 ms blank screen, four squares were presented with the fixation cross for 500 ms. Then another 500-ms blank was shown, followed by the simultaneously presented two scene images for 27 ms or 507 ms. After presentation, two mask images were on for 1,000 ms. Then the response cue followed. Participants pressed the “F” key if they judged the two images belonged to the same basic-level category or “J” key if not. They were encouraged to respond as accurately as possible. After a 500-ms delay, a black square was presented in one of the eight possible positions together with the fixation cross. Participants were required to indicate whether the position matched one of the previous positions. Participants pressed the “F” key if they thought the presented stimulus matched with memorized stimulus or the “J” key if not. After a 500-ms blank, a capital letter was presented and participants were required to judge whether the presented letter matched one of the four letters that had been repeated during the trial or not. If the letters matched, then pressed “F” key, if not, pressed “J” key. The trial ended after the keypress. In the single task condition, the participants were only required to accomplish articulatory suppression task and scene gist discrimination task. The procedure of each trial was similar to that of dual task. The only difference was that no memory arrays and memory test stimuli were shown in the single task condition.

In the scene gist discrimination task, during half of the trials, two different scene images presented simultaneously belonged to the same basic-level categories (e.g., two images of different mountains); during the remaining half of trials, scene images belonged to different basic-level categories (e.g., mountain vs. street). In addition, under the two conditions, half of the trials in each condition were dual tasks, and the other half were single task. The learning phase included 80 trials, and each basic-level category had 10 trials. After the end of the exercise phase, which included 24 trials, the experiment phase began which included 672 trials. Among these trials, each superordinate level pairing condition contained 224 trials (each basic-level pairing condition had 28 trials). Each presentation time condition and each working memory load condition all had 336 trials. Participants had a break for 1 min after finishing 112 trials and pressed “Q” to continue.

### Results

The trials in which the participants did not correctly perform the working memory task were excluded (2,065 trials, 19.21%). We used the remaining trials (trials of single task and trials of dual tasks) to analyze participants' responses on the difference between same or different basic-level images by employing the nonparametric signal detection measure *A*' of sensitivity. Similar to the general signal detection measure *d*' of sensitivity, *A*' would exclude the possibility that the results were affected by some certain biases in participants' responses (Grier, 1971).

Descriptive statistics of *A*' in the different conditions in Experiment 1 are shown in Table 2. A 3 (Scene Pairing: natural–natural, manmade–manmade, manmade–natural) × 2 (Presentation Time: 27 ms vs. 507 ms) × 2 (working memory load: no-load vs. four-load) repeated-measures analysis of variance (ANOVA) was conducted. The main effect of presentation time was significant, *F*(1, 15) = 52.00, *p* < .001, $\eta_{\text{p}}^{2}$ = 0.78, and *A*' with 507 ms (*M* = 0.77, *SE* = 0.02) was higher than that with 27 ms (*M* = 0.54, *SE* = 0.03). The main effect of scene pairing was also significant, *F*(2, 30) = 23.49, *p* < .001, $\eta_{\text{p}}^{2}$ = 0.61, and Bonferroni adjustment (To maintain an error rate of α = .05, we adjusted the critical *p* value to α = .0168) indicated that *A*' with manmade–natural (*M* = 0.75, *SE* = 0.02) was higher than that with manmade–manmade (*M* = 0.54, *SE* = 0.03, *p* < .001). The main effect of working memory load was also significant, *F*(1, 15) = 8.47, *p* = .011, $\eta_{\text{p}}^{2}$ = 0.36, *A*' with no-load (*M* = 0.69, *SE* = 0.02) was higher than that with four-load (*M* = 0.62, *SE* = 0.03).

**Table 2.** Descriptive Statistics Table of *A*' with Different Conditions in Experiment 1.

Scene pairing		Manmade–manmade				Natural–natural				Manmade–manmade			
Working memory load		No-load		Four-load		No-load		Four-load		No-load		Four-load	
Presentation time (ms)		27	507	27	507	27	507	27	507	27	507	27	507
Experiment 1	Mean	0.61	0.63	0.31	0.62	0.60	0.79	0.49	0.83	0.64	0.86	0.60	0.89
	*SD*	0.10	0.25	0.28	0.31	0.23	0.11	0.23	0.23	0.09	0.10	0.17	0.06

**Figure 3.**
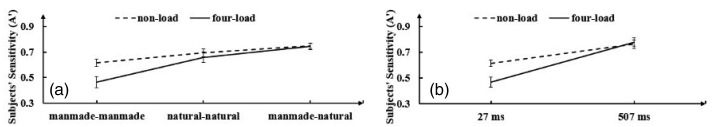
Mean subject sensitivity (*A*') as a function of scene pairing, spatial working memory load, and presentation time in Experiment 1. Error bars are standard errors of the means in subplot. (a) Mean subjects' sensitivity (*A*') as a function of scene pairing and spatial working memory load and (b) Mean subjects' sensitivity (*A*') as a function of spatial working memory load and presentation time.

The interaction between working memory load and scene pairing was significant (see Figure 3(a)), *F*(2, 30) = 3.72, *p* = .036, $\eta_{\text{p}}^{2}$ = 0.20. A simple effect analysis revealed that *A*' with manmade–manmade pairing (*M* = 0.62, *SE* = 0.03) under no-load condition was larger than that (*M* = 0.46, *SE* = 0.04) under four-load condition (*p* = .008), and no significant effect was found between *A*'s with manmade–natural and natural–natural pairings (*M* = 0.75; *M* = 0.69) under no-load condition and those (*M* = 0.75; *M* = 0.66) under four-load condition (*p* = .809, *p* = .394).

The interaction between working memory load and presentation time was also significant (see Figure 3(b)), *F*(1, 15) = 6.65, *p* = .021, $\eta_{\text{p}}^{2}$ = 0.31. And simple effect analysis revealed that when the presentation time was 27 ms, *A*' with no-load (*M* = 0.61, *SE* = 0.03) was higher than that with four-load (*M* = 0.47, *SE* = 0.04), *F*(1, 15) = 14.02, *p* = .002, $\eta_{\text{p}}^{2}$ = 0.48, but when the presentation time was 507 ms, the difference of *A*'s between no-load (*M* = 0.76, *SE* = 0.03) and four-load (*M* = 0.78, *SE* = 0.03) was not significant, *F*(1, 15) = 0.22, *p* = .646, $\eta_{\text{p}}^{2}$ = 0.01.

The above analysis revealed that spatial working memory load had an effect on scene gist discrimination. Next, we use MDS borrowed from the study of Kadar and Ben-Shahar (2012) to explore the order of hierarchical processing in scene gist recognition. First, we calculated *A*'s of all pairs of scene categories (Table 3). It was evident that the ability of participants to discriminate between different categories was different. For example, participants had better performance in discriminating streets from coasts (0.78) and tall building from forests (0.82). However, performance dropped considerably when participants discriminated open country from mountains (0.59), streets from inside cities (0.67). In a sense, the sensitivity for the different pairs of categories could be interpreted as the perceptual distance between these pairs of categories. Lower sensitivity means that the categories share more common features, it is difficult to discriminate the categories, and the perceptual distance is lower. Conversely, higher sensitivity implies that the perceptual distance is higher and it is easy to discriminate the categories (Kadar & Ben-Shahar, 2012). With this definition in mind, we adopted MDS to make a further analysis to obtain the structure of the perceptual space of different categories.

**Table 3.** Perceptual Data Matrix obtained by Measuring Participants' Average Sensitivity Between all Pairs of Scene Categories of Experiment 1.

		Coast	Forest	Highway	Inside city	Mountain	Open country	Street	Tall building
No-load	Coast	0.74	0.76	0.71	0.77	0.66	0.69	0.78	0.80
	Forest	–	0.75	0.76	0.76	0.70	0.69	0.77	0.82
	Highway	–	–	0.73	0.76	0.80	0.74	0.64	0.79
	Inside city	–	–	–	0.76	0.73	0.77	0.67	0.63
	Mountain	–	–	–	–	0.77	0.59	0.79	0.77
	Open country	–	–	–	–	–	0.67	0.77	0.79
	Street	–	–	–	–	–	–	0.77	0.64
	Tall building	–	–	–	–	–	–	–	0.79
Four-load	Coast	0.73	0.72	0.67	0.79	0.57	0.54	0.77	0.82
	Forest	–	0.77	0.80	0.82	0.62	0.56	0.70	0.85
	Highway	–	–	0.74	0.75	0.73	0.69	0.57	0.73
	Inside city	–	–	–	0.74	0.65	0.69	0.50	0.55
	Mountain	–	–	–	–	0.80	0.59	0.76	0.83
	Open country	–	–	–	–	–	0.72	0.82	0.65
	Street	–	–	–	–	–	–	0.79	0.61
	Tall building	–	–	–	–	–	–	–	0.80

**Figure 4.**
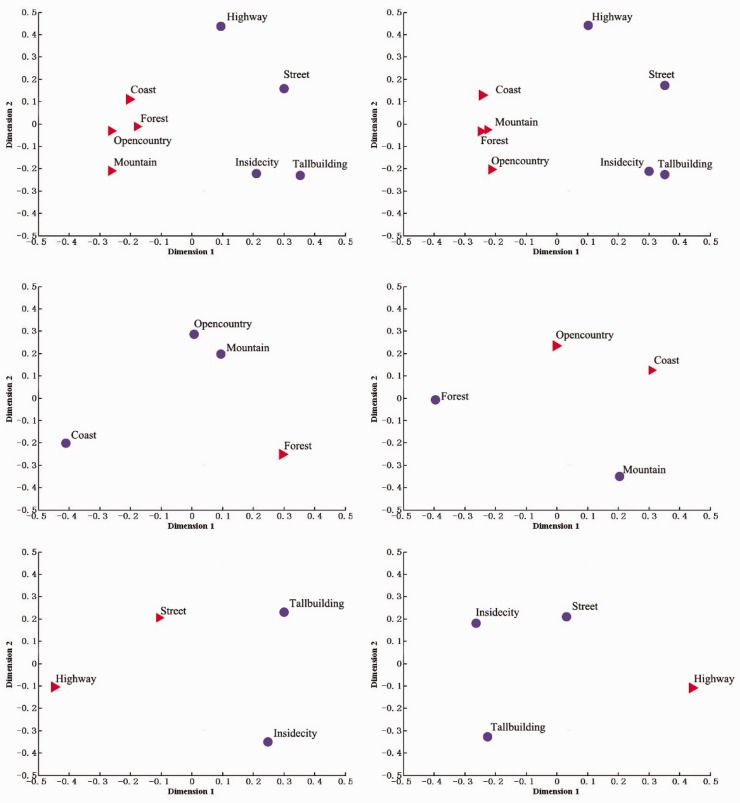
Applying MDS and clustering analyses of three diffent levels of the scene categorization hierarchy. (a) MDS and clustering analyses of the first level of the scene categorization hierarchy, and results on the eight scene categories can be interpreted as division between natural (four red triangles) and manmade (four blue dots) scenes. (b) MDS and clustering analyses of the second level of the scene categorization hierarchy and results on the four natural scene categories can be interpreted as division between coast, open country, mountain (three blue dots) and forest (one red triangle) or between coast, open country (two red triangles) and mountain, forest (two blue dots). (c) MDS and clustering analyses of the second level of the scene categorization hierarchy and results on the four manmade scene categories can be interpreted as division between highway, street (two red triangles) and tall building, Inside city (two blue dots) or between tall building, street, Inside city (three blue dots) and highway (one red triangle).

MDS analysis revealed that participants first divided scene images into two clusters (see Figure 4(a)), natural (left, red triangles) and manmade (right, blue dots), regardless of whether there was working memory load or not. Then, we used MDS analysis for basic level (natural–natural, manmade–manmade; see Figure 4(b) and (c)). The results revealed that under the no-load condition, participants discriminated forests from the other three natural scenes, highway and streets from inside cities, and tall buildings; but under the four-load condition, participants would have a tendency to separate forests and mountains from open country and coasts, highway from the other three manmade scenes.

### Discussion

Two significant interactions were found. The interaction between scene pairing and spatial working memory load revealed that spatial working memory load (position-based working memory load) mainly affected the discrimination of different basic-level pairing, especially on discrimination of manmade–manmade pairing (e.g., Inside city vs. highway), hardly or did not affect the discrimination of different superordinate level pairing (manmade–natural pairing; e.g., Inside city vs. Coast). This finding suggested that spatial working memory load had less influence on superordinate level processing than on basic-level processing, supporting the hypothesis that the superordinate level has priority.

The interaction between spatial working memory load and presentation time revealed that spatial working memory load had a larger influence on early than late processing of scene gist discrimination, which suggests that spatial information contributes to scene gist discrimination at early processing stages.

Furthermore, MDS analysis revealed that regardless whether there was spatial working memory load or not, participants would discriminate scene categories on superordinate level first and then on the basic level. Based on these results, we concluded that spatial working memory load did not affect the hierarchical processing order of scene gist recognition.

To sum up, Experiment 1 mainly found that spatial working memory load mainly influenced basic-level scene gist discrimination and this influence happens at early processing stages, supporting processing priority of the superordinate level.

## Experiment 2: Visual Object Working Memory Load

In this experiment, we replaced the spatial working memory load with visual object working memory load to examine the effects of object working memory load on hierarchy in scene gist processing.

### Methods

#### Participants

After giving informed consent, a group of 16 participants (15 females, one male, average age = 21.13) were paid to take part in Experiment 2. All had normal or corrected to normal vision, and all were naïve about the purpose of this experiment.

#### Apparatus, stimuli, design, and procedure

**Figure 5.**
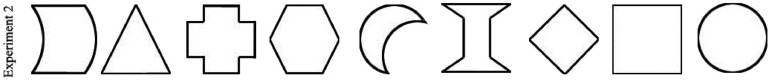
Stimuli of Experiment 2 for visual object working memory task.

The methods in Experiment 2 were as in Experiment 1 with the following exception: Instead of the spatial working memory task, a visual object working memory was administered. Out of the eight possible geometric figures (Figure 5, size: 2.26° × 2.26° of visual angle), four were shown on each trial and observers were asked to memorize them and indicate whether a probe figure corresponded to one of the memorized figures.

### Results

The preprocessing of data in this experiment was the same as in Experiment 1. We excluded the trials of the dual task in which the working memory task was not correctly performed. In this experiment, a total of 2,180 trials (20.28%) were excluded. We used the remaining trials to analyze participants' responses on the difference between same or different basic-level images by employing the nonparametric signal detection measure *A*' of sensitivity (Grier, 1971).

Descriptive statistics of *A*'s of different conditions in Experiment 2 are shown in Table 4. A 3 (Scene Pairing: natural–natural, manmade–manmade, manmade–natural) × 2 (Presentation Time: 27 ms vs. 507 ms) × 2 (Object Working Memory Load: no-load vs. four-load) repeated-measures ANOVA was conducted. The main effect of presentation time was significant, *F*(1, 15) = 9.61, *p* = .007, $\eta_{\text{p}}^{2}$ = 0.39, and *A*' with 507 ms (*M* = 0.75, *SE* = 0.02) was higher than that with 27 ms (*M* = 0.64, *SE* = 0.03). The main effect of scene pairing was also significant, *F*(2, 30) = 37.24, *p*<.001, $\eta_{\text{p}}^{2}$ = 0.73, and Bonferroni adjustment (To maintain an error rate of α = .05, we adjusted the critical *p* value to α = .0168.) indicated that *A*' with manmade–natural (*M* = 0.79, *SE* = 0.02) was higher than that with manmade–manmade (*M* = 0.55, *SE* = 0.03, *p* < .001). The main effect of working memory load was also significant, *F*(1, 15) = 14.13, *p* = .002, $\eta_{\text{p}}^{2}$ = 0.49, *A*' with no-load (*M* = 0.72, *SE* = 0.02) was higher than that with four-load (*M* = 0.67, *SE* = 0.02).

**Table 4.** Descriptive Statistics Table of *A*'s of Different Conditions in Experiment 2.

Scene pairing		Manmade–manmade				Natural–natural				Manmade–manmade			
Working memory load		No-load		Four-load		No-load		Four-load		No-load		Four-load	
Presentation time (ms)		27	507	27	507	27	507	27	507	27	507	27	507
Experiment 2	Mean	0.60	0.67	0.46	0.48	0.70	0.78	0.68	0.79	0.69	0.89	0.69	0.90
	*SD*	0.15	0.24	0.31	0.33	0.21	0.16	0.21	0.20	0.10	0.05	0.12	0.07

**Figure 6.**
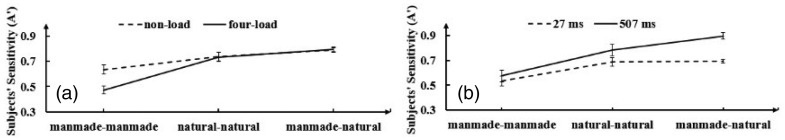
Mean subject sensitivity (*A*') as a function of scene pairing, object working memory load, and presentation time in Experiment 2. Error bars are standard errors of the means in subplot. (a) Mean subjects' sensitivity (*A*') as a function of scene pairing and object working memory load and (b) Mean subjects' sensitivity (*A*') as a function of scene pairing and presentation time.

The interaction between scene pairing and object working memory load was significant (see Figure 6(a)), *F*(2, 30) = 8.18, *p* = .001, $\eta_{\text{p}}^{2}$ = 0.35. A simple effect analysis revealed that the *A*' with manmade–manmade pairing under no-load condition (*M* = 0.63) was larger than that under four-load condition (*M* = 0.47, *p* = .001), and no significant effect was found between the *A*'s with manmade–natural and natural–natural pairings under no-load condition (*M* = 0.79; *M* = 0.74) and those under four-load condition (*M* = 0.80; *M* = 0.74, *p* = .619, *p* = .974).

The interaction between scene pairing and presentation time was also significant (see Figure 6(b)), *F*(2, 30) = 4.91, *p* = .014, $\eta_{\text{p}}^{2}$ = 0.25. Simple effect analysis revealed that *A*' with manmade–natural pairing was larger when the presentation time was 507 ms (*M* = 0.90) than that when the presentation time was 27 ms (*M* = 0.69, *p* < .001); *A*' with natural–natural pairing was marginally larger when the presentation time was 507 ms (*M* = 0.78) than that when the presentation time was 27 ms (*M* = 0.69, *p* = .067); and no significant effect was found between the *A*' with manmade–manmade pairings when the presentation time was 507 ms (*M* = 0.57) and 27 ms (*M* = 0.53, *p* = .523).

The results of MDS analysis (Table 5) in this experiment were the same as those of Experiment 1, participants clustered scene images on superordinate level first and then on the basic level.

**Table 5.** Perceptual Data Matrix Obtained by Measuring Participants' Average Sensitivity Between All Pairs of Scene Categories of Experiment 2.

		Coast	Forest	Highway	Inside city	Mountain	Open country	Street	Tall building
No-load	Coast	0.68	0.75	0.78	0.77	0.78	0.73	0.79	0.85
	Forest	–	0.72	0.79	0.81	0.72	0.66	0.83	0.80
	Highway	–	–	0.72	0.76	0.83	0.74	0.69	0.72
	Inside city	–	–	–	0.73	0.86	0.85	0.69	0.57
	Mountain	–	–	–	–	0.73	0.67	0.80	0.82
	Open country	–	–	–	–	–	0.66	0.84	0.83
	Street	–	–	–	–	–	–	0.74	0.76
	Tall building	–	–	–	–	–	–	–	0.78
Four-load	Coast	0.72	0.79	0.78	0.79	0.79	0.69	0.76	0.79
	Forest	–	0.73	0.78	0.76	0.68	0.66	0.85	0.77
	Highway	–	–	0.73	0.81	0.77	0.81	0.60	0.84
	Inside city	–	–	–	0.79	0.89	0.78	0.46	0.63
	Mountain	–	–	–	–	0.76	0.63	0.74	0.86
	Open country	–	–	–	–	–	0.70	0.80	0.79
	Street	–	–	–	–	–	–	0.79	0.72
	Tall building	–	–	–	–	–	–	–	0.80

### Discussion

Similar to Experiment 1, this experiment found two significant interactions. The interaction between scene pairing and object working memory load revealed that object working memory load (shape-based working memory load) mainly affected the discrimination of different basic-level pairings, especially on the discrimination of manmade–manmade pairing, hardly or did not affect the discrimination of different superordinate level pairings. And this finding suggested that object working memory load had less influence on superordinate level processing than on basic-level processing, supporting superordinate level processing priority hypothesis.

The interaction between scene pairing and presentation time revealed that presentation time had a larger influence on discrimination of different superordinate level scene pairings than different basic-level scene pairing, which suggested that object information mainly contributes to superordinate level scene gist discrimination at later stages.

To sum up, Experiment 2 found that object working memory load mainly influence the basic-level scene gist processing and this influence happens at later processing stages, supporting superordinate level processing priority.

## General Discussion

In this study, we designed two experiments by combining a perceptual discrimination task and a visuospatial working memory task to investigate the effects of visuospatial working memory load on hierarchy of scene gist processing. The four important findings are as follows.

Interaction between scene pairing and spatial/object working memory load. Although different types of working memory (spatial working memory load and object working memory load) were employed in Experiments 1 and 2, the findings from these two experiments were similar, revealing significant interactions between scene pairing and spatial/object working memory load. Further analysis showed that spatial/object working memory load mainly influenced discrimination of different basic-level scene pairings (manmade–manmade pairing; e.g., Inside city vs. Highway), rather than superordinate level scene pairings (manmade–natural pairing; e.g., Inside city vs. Coast). This finding suggests that superordinate level scene category processing has priority over basic-level scene category processing, supporting the superordinate level processing advantage. These findings are consistent with recent studies (Kadar & Ben-Shahar, 2012; Malcolm et al., 2014; Ramkumar et al., 2014; Wu et al., 2014).

The second finding is that the MDS analyses in Experiments 1 and 2 revealed superordinate level processing advantage. This finding showed that participants clustered scene categories on superordinate level first, then clustered scene categories on the basic level, regardless of working memory load or whether the load was spatial or nonspatial (object), supporting superordinate level processing advantage, consistent with recent studies (Kadar & Ben-Shahar, 2012; Malcolm et al., 2014).

The third finding is the interaction between spatial working memory load and presentation time in Experiment 1 (see Figure 3(b)). This interaction showed that spatial working memory load had a larger influence on early than later processing stages of scene gist discrimination, which suggested that spatial information contributes to scene gist discrimination at earlier processing stages.

The fourth finding is the interaction between scene pairing and presentation time in Experiment 2 (see Figure 6(b)). This interaction showed that presentation time had a larger influence on discrimination of different superordinate level scene pairings than different basic-level scene pairings, which suggested that the object information contained in superordinate level scene paring has priority over that contained in basic-level scene pairings in the later processing stages.

The former two findings have the same patterns in Experiments 1 and 2, convergently supporting superordinate level processing priority. However, the latter two findings may reflect different roles of spatial and object information at different processing stages in scene gist recognition: Spatial information contributes to the earlier stages in scene gist recognition and object information contributes to the later stages in scene gist recognition. Further studies are required to clarify this issue.

**Figure 7.**
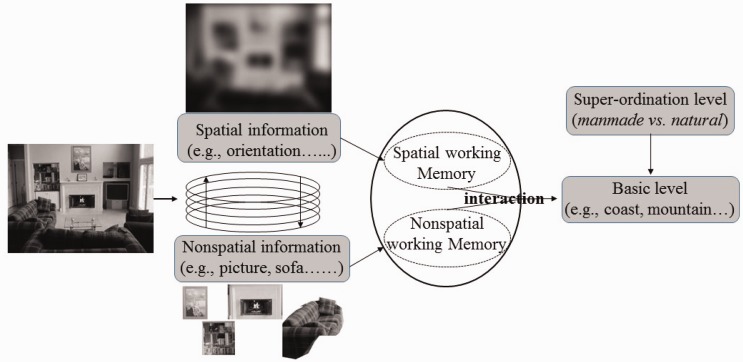
The interaction model of scene gist recognition and visuospatial working memory.

In summary, the innovation of present study was that we investigated the extracting mechanism of object and spatial information during scene gist recognition by manipulating spatial and object working memory loads. The results showed that scene gist recognition would share some common spatial and object working memory resources. Based on previous findings and present findings, we proposed the interaction model of visuospatial working memory and scene gist recognition (see Figure 7): Spatial and object working memory both have effects on scene gist recognition, but mainly on the basic level, rather than on the superordinate level. But on the other hand, object and spatial information that we engaged to cluster scene images are learned during our daily lives, and this information is stored in long-term memory. For example, we make the association between shoes and shoe shop, trees, and forest. Therefore, subsequent studies should consider the interaction between working memory and long-term memory. To this end, we could use event-related potentials technique to record the brain activity during scene gist recognition. Previous investigators found that if one cognitive task occupied working memory resources, then the contralateral delay activity (CDA) is activated in LOC; and if it occupied long-term memory resources, P1 or P170 component is induced in prefrontal lobe (Carlisle, Arita, Pardo, & Woodman, 2011; Woodman, Carlisle, & Reinhart, 2013). Recording and analyzing contralateral delay activity and P170 could reveal the interaction mechanism between working memory and long-term memory. In addition, the present study just considered the no-load and four-load conditions, the subsequent studies could also adopt the three-load, two-load or one-load condition to investigate the effect of visuospatial working memory capacity on scene gist recognition.

## Conclusions

In summary, we reach the following conclusions: (a) spatial load and object load affected gist recognition and most likely has stronger effects on the basic level rather than on the superordinate level, supporting an advantage of superordinate level processing, consistent with an MSD analysis; (b) spatial load has a larger impact on discrimination of scene pairings at early stages than at later stages; object load has a larger influence on that at later stages than at early stages; and both of them have different roles in scene gist recognition.
